# Orthograde Apical Barrier in Non-Vital Immature Permanent Teeth: A Narrative Review of Clinical Pathways, Procedural Determinants, and Evidence Gaps

**DOI:** 10.3390/dj14080509

**Published:** 2026-08-11

**Authors:** Yasser Alsayed Tolibah, Nada Bshara, Osama Aljabban, Chaza Kouchaji, Thuraya Lazkani, Mohammad Tamer Abbara, Marwan Alhaji, Ziad D. Baghdadi

**Affiliations:** 1 Department of Pediatric Dentistry, Damascus University, Damascus P.O. Box 3062, Syria; yasseralsayedtolibah@gmail.com (Y.A.T.); gmmn2012@gmail.com (N.B.); shazako@yahoo.com (C.K.); mrwan.2013.55.a@gmail.com (M.A.); 2 Department of Endodontics, Damascus University, Damascus P.O. Box 3062, Syria; dr.ossamaljabban@gmail.com (O.A.); dr.thuraya1979@gmail.com (T.L.); 3 Department of Endodontics, University of Kalamoon, Damascus P.O. Box 3062, Syria; tamerabbara@gmail.com; 4 TopSmiles Pediatric Dentistry & Orthodontics, 246 St. Anne’s Road, Winnipeg, MB R2M 3A4, Canada; 5 Butterfly Dental Group, Winnipeg, MB R3Y 1P5, Canada; 6 Children’s Dental Centre, 1630 Ness Ave., Winnipeg, MB R3J 3X1, Canada

**Keywords:** immature permanent teeth, apical barrier, pulp necrosis, pediatric endodontics

## Abstract

**Background/Objectives:** Pulp necrosis in immature permanent teeth arrests root development, leaving an open apex, thin, divergent dentinal walls, and an unfavorable crown-to-root ratio that predisposes the tooth to fracture and complicates endodontic management. An apical barrier using hydraulic calcium silicate cements has become the first-line orthograde approach for these teeth when regenerative procedures are not indicated or feasible. This narrative review synthesizes current evidence on the complete clinical pathway for apical barrier placement in immature permanent teeth, with particular emphasis on the procedural determinants of barrier formation. It also critically appraises where the evidence is robust and where it remains uncertain. **Methods**: The relevant English-language literature on root development, the etiology of pulp necrosis in immature teeth, diagnosis, isolation, apical barrier methods, calcium silicate materials, and treatment outcomes was reviewed through targeted searches of PubMed/MEDLINE, Scopus, Web of Science, the Cochrane Library, and Google Scholar through June 2026. The literature search was completed in June 2026; therefore, studies published after this date were not included. Evidence was narratively synthesized according to the clinical sequence of diagnosis, treatment selection, isolation, disinfection, barrier formation, restoration, follow-up, and evidence gaps. Throughout, an explicit distinction was maintained between clinical and laboratory evidence. **Results:** Sensibility testing is widely considered unreliable in immature teeth, complicating diagnosis. Isolation is often challenging because of traumatic crown loss. Successful treatment depends on adequate chemical disinfection, judicious minimal instrumentation, a well-condensed apical barrier of at least 4–5 mm, and a definitive coronal seal that also addresses the weak cervical dentin. Calcium silicate cements—principally MTA, Biodentine, and pre-mixed bioceramic putties—achieve high clinical success. Observational evidence further suggests, as a hypothesis requiring prospective confirmation, that material choice may be less decisive than operator experience and the quality of the coronal restoration. **Conclusions:** Apical barrier placement with hydraulic calcium silicate cements is a predictable orthograde preservation approach for non-vital immature permanent teeth, particularly when regenerative endodontic procedures are not indicated, not feasible, or unlikely to yield predictable clinical outcomes. Well-designed randomized clinical trials with standardized reporting are needed.

## 1. Introduction

The permanent dentition can be rendered non-vital before root formation is complete, most often as a consequence of dental trauma, untreated caries or developmental dental anomalies in children and adolescents [1,2].

When the pulp becomes necrotic in such an immature tooth, the function of Hertwig’s epithelial root sheath is disrupted and root development ceases, leaving a tooth with a short root, thin and divergent dentinal walls, a wide root canal and an open (often blunderbuss) apex [1]. This anatomical configuration produces an unfavourable crown-to-root ratio and markedly reduced resistance to fracture, while the absence of an apical constriction makes conventional obturation, working-length determination and effective disinfection particularly demanding [3].

For decades, the management of these teeth relied on long-term calcium hydroxide apexification to induce a calcified apical barrier. Although effective in forming a barrier, this approach requires many months and multiple visits and has been associated with a time-dependent weakening of radicular dentine and a clinically relevant risk of cervical root fracture [4]. The introduction of hydraulic calcium silicate cements as artificial apical barriers transformed this field, enabling single- or two-visit creation of an apical barrier, immediate obturation and reported success rates broadly comparable or superior to those of calcium hydroxide [5,6]. More recently, regenerative endodontic procedures have emerged as a biologically attractive alternative that may permit continued root maturation; however, the longer-term and comparative evidence base remains incomplete. Apical barrier treatment remains a well-established orthograde option, particularly when revascularisation is not indicated or not feasible; the choice between the two, however, is not fixed and depends on the stage of root development, restorability, infection control, patient cooperation, clinician experience, and the potential for continued root maturation [7,8].

Despite its popularity, the literature on the apical barrier technique is heterogeneous: individual studies tend to address a single variable—material type, barrier thickness, irrigation, condensation method, or outcome—rather than the complete clinical pathway. Several procedural steps that are specific to the immature tooth are frequently under-reported. This review is guided by a central clinical question: Across the complete orthograde treatment pathway for non-vital immature permanent teeth, which procedural determinants most plausibly influence the clinical and radiographic outcome of apical-plug therapy, and how robust is the evidence underlying each? To address this, we integrate the contemporary evidence along the full clinical sequence—diagnosis, isolation, disinfection, the procedural determinants of plug formation, material selection, the definitive restoration, and patient- and operator-related factors—and identify the questions that remain unresolved at each step.

Two boundaries define the scope. First, the review concerns the orthograde apical-plug (apexification) pathway in non-vital immature permanent teeth; regenerative endodontic procedures are considered only where a comparative decision is required, and vital pulp therapy only as indirect supporting evidence. Second, throughout the synthesis we distinguish explicitly among established clinical recommendations, emerging clinical evidence, indirect evidence (from mature teeth or laboratory models), expert opinion, and speculative proposals, so that the strength of each statement is transparent to the reader.

In contemporary pediatric dentistry, apical barrier therapy should be understood not merely as apexification with a newer material, but as a structured decision pathway that preserves compromised immature permanent teeth when regenerative treatment is not appropriate, while requiring accurate diagnosis, procedural skill, definitive restoration, and equitable access to follow-up care.

## 2. Methods of the Narrative Review

This narrative review was developed through targeted searches of PubMed/MEDLINE, Scopus, Web of Science, the Cochrane Library, and Google Scholar up to June 2026. Search terms combined: “immature permanent teeth,” “open apex,” “apexification,” “apical barrier,” “mineral trioxide aggregate,” “MTA,” “Biodentine,” “bioceramic putty,” “calcium silicate cement,” “regenerative endodontics,” “pediatric endodontics,” “dental trauma,” “irrigation,” “intracanal medicament,” “rubber dam isolation,” “working length,” “CBCT,” “fibre post,” and “coronal restoration.” English-language clinical studies, randomised trials, systematic reviews, guidelines, laboratory studies, and relevant case reports were considered when they informed the clinical pathway or the procedural determinants of apical barrier therapy.

Searches combined these terms using the Boolean operators “AND” and “OR” and were adapted to the syntax and field tags of each database. The search covered literature published up to June 2026, with no lower date restriction, encompassing both foundational and recent studies. Records were identified primarily through database searches, supplemented by screening the reference lists of relevant articles to locate additional sources not retrieved electronically. Titles and abstracts were screened for relevance to the apical-barrier pathway in non-vital immature permanent teeth, and potentially relevant full texts were then examined. Given the narrative design, screening and final source selection were performed by the lead author in consultation with the co-authors, rather than through independent duplicate screening. No formal record count or flow diagram was maintained. Studies were excluded if they did not address the immature permanent tooth, if the procedural detail relevant to the treatment pathway could not be extracted, or if they duplicated findings already represented by stronger evidence.

Because this was conceived as a narrative rather than a systematic review, no comprehensive database protocol, formal meta-analysis, or structured risk-of-bias assessment was undertaken, and the synthesis is therefore subject to the selection and interpretive limitations inherent to this design.

Throughout, an explicit distinction was maintained between evidence derived from clinical studies and that derived from laboratory (in vitro), ex vivo, or case-report sources, and the strength of any procedural statement was deliberately calibrated to the nature of its supporting evidence. Where recommendations acquire a quantitative or comparative character—such as specific barrier thicknesses or material rankings—they should be read as pragmatic suggestions grounded in the best currently available evidence, frequently of laboratory origin, rather than as formal clinical guidelines; corroboration by well-designed randomised clinical trials remains necessary.

When multiple studies addressed the same question, priority was given to the evidence type most appropriate to that question: Clinical outcomes were based, where available, on clinical trials, systematic reviews, and guidelines, whereas laboratory and ex vivo studies were relied upon mainly for mechanistic or procedural questions (such as sealing ability or fracture resistance) for which direct clinical data were lacking. Case reports were used illustratively rather than as a basis for recommendations. Where findings were contradictory, the discrepancy was reported explicitly in the text rather than resolved in favor of a single result, and the difference was attributed, where possible, to the nature of the evidence (for example, in vitro versus clinical) or to methodological heterogeneity among studies.

Evidence was narratively synthesised according to the clinical sequence of diagnosis, treatment selection, isolation, disinfection, barrier formation, restoration, follow-up, and evidence gaps.

## 3. Management of Non-Vital Immature Permanent Teeth by Apexification with Apical Barrier

### 3.1. Indication

Apexification is indicated as one of the most widely accepted therapeutic approaches for the management of non-vital immature permanent teeth (with a Cvek root-development stage of II to IV) with or without a periapical lesion [9,10] to form a calcified barrier at the apex to ensure a tight seal of the root canal, prevent overfilling and promote healing of the periapical tissues [11,12].

The dental literature lacks clear criteria for the apical dimension at which a tooth is considered to have an open apex; this depends largely on the criteria used in individual controlled randomized clinical studies. Recent studies have considered the minimum dimension at which a tooth is classified as immature to range between 0.6 and 1 mm, depending on tooth type (the range narrows posteriorly) and on the intended type of treatment (regenerative procedures require larger dimensions). Some studies relied on the radiographic appearance of the tooth itself, considering it immature if its root was shorter than expected, its walls were divergent, or the apical lumen was wider than the canal lumen [6,9,10,13,14,15,16].

### 3.2. Calcium Hydroxide Apexification

The use of calcium hydroxide [Ca(OH)_2_] paste as a repeated, long-term intracanal dressing was observed to induce apical closure [17]. The alkalinity of calcium hydroxide stimulates the stem cells of the periapical papilla to transform into cementum- and dentine-forming cells, and apical formation continues through the deposition of hard tissue in the apical region in cases where the teeth are asymptomatic with radiographic signs indicating closure of the apical foramen [18,19]. This technique is distinguished by its excellent ability to disinfect the root canal owing to the high alkalinity of calcium hydroxide [4,20]. However, it has drawbacks, namely the need for multiple treatment visits over a long period of between 9 and 24 months [21], as well as a decrease in fracture resistance over time at the level of the pre-cervical dentine (the mechanically weakest region in immature teeth) because of the reaction of calcium hydroxide with dentine and the dissolution of its organic components, which alters the nature of the acidic proteins and proteoglycans that act in dentine as binding agents between the collagen network and hydroxyapatite crystals, rendering the dentine more brittle and potentially leading to cervical root fracture and the risk of complete tooth loss [4,22]. Immature permanent teeth with a Cvek root-development stage of I have been identified as a negative prognostic factor for such fractures when treated with calcium hydroxide dressings, with fracture rates ranging from 77% in the least mature teeth to 27% in the more mature teeth; the deficiency of root development was observed to be more influential than the duration of calcium hydroxide dressing use [10,23].

### 3.3. Apical Barriers

Another effective technique was developed as an alternative to calcium hydroxide apexification, working to induce the formation of apical hard tissue with the aid of an artificial barrier at the apex to assist closure of the root canal in teeth with open apices—known as the apical barrier, using calcium silicate materials.

Calcium silicate cements have been distinguished from conventional calcium hydroxide dressings by numerous advantages, such as enhanced compressive strength, reduced solubility after setting, improved sealing ability to prevent bacterial ingress, antibacterial activity against facultative anaerobes, an excellent capacity to induce the formation of mineralised tissue, and a positive biological interaction with the surrounding dental tissues, all of which remain unaffected by contact with tissue fluids or blood [24,25,26]. However, these materials also have some limitations, such as high cost, difficulty of mixing and handling, the potential to cause tooth discolouration, a long setting time, and the considerable waste associated with materials available in capsule form [27,28,29].

This technique yields excellent results in periapical tissue healing and radiographic signs indicating closure of the apical foramen [6,30]. It is distinguished by the immediate closure of the open apex, which substantially reduces treatment duration, in addition to its excellent biocompatibility arising from the use of highly biocompatible calcium silicate cements, which improves their interaction with the periapical tissues by stimulating cell proliferation and differentiation [6,15,31].

### 3.4. The Practical Stages of Apical Barrier Formation

#### 3.4.1. Diagnostic Considerations and the Limitations of Pulp Testing

Establishing a correct pulpal diagnosis is the prerequisite that determines whether a tooth is a candidate for apical barrier at all, yet this is uniquely difficult in immature teeth. Conventional thermal and electric sensibility tests are widely regarded as unreliable in immature permanent teeth, principally because the myelinated nerve fibres of the pulp—and the plexus of Raschkow at the pulpo-dentinal junction—are not fully developed until several years after eruption [32]. Consequently, a vital but immature tooth may fail to respond to the electric pulp test, producing false-negative results, while the subjective nature of these tests and the limited cooperation of young patients further reduce their diagnostic accuracy [33]. This limitation is compounded after trauma, where sensory function may be temporarily lost even when the vascular supply is intact [34].

Because sensibility tests assess innervation rather than blood supply, they do not reflect the true vitality of the pulp. For this reason, a diagnosis of pulp necrosis should not be based on a single test, but should integrate the history, clinical examination, multiple special tests and radiographic findings [35]. This is especially important in immature teeth following trauma, where revascularisation of the pulp remains possible and a premature decision to intervene endodontically would needlessly arrest root development. Vitality-based methods that assess pulpal blood flow rather than innervation—such as pulse oximetry and laser Doppler flowmetry, and more recently transmitted-light plethysmography—have shown encouraging diagnostic accuracy in young permanent incisors and may, in time, offer a more reliable basis for decision-making in this group, although they are not yet routine [36]. In practice, serial monitoring and confirmation of necrosis by unambiguous clinical or radiographic signs of infection remain the most defensible approach before committing an immature tooth to apical barrier.

Radiographic examination complements sensibility testing and is indispensable for staging root development, locating the apex, and assessing the periapical tissues. Conventional two-dimensional periapical radiographs remain the first-line and routine modality, yet their diagnostic yield in the immature, frequently traumatized tooth is limited: superimposition of anatomical structures and the two-dimensional compression of a three-dimensional object mean that early periapical lesions, root fractures, and resorptive defects on the buccal or palatal surfaces may go undetected, and a substantial proportion of cases show clinical signs without corresponding radiographic evidence. Cone-beam computed tomography (CBCT) can overcome many of these limitations by providing three-dimensional, high-resolution visualization of the root, the open apex, and the surrounding bone. When conventional imaging is insufficient and the result is expected to alter management, CBCT may be particularly valuable in this group for determining the true extent of the periapical lesion, detecting concomitant root fractures after trauma, characterizing and monitoring external and internal inflammatory resorption, and, in selected cases, estimating the thickness of the residual dentinal walls. When such three-dimensional information is obtained, it may help inform the choice among apexification, regenerative treatment, and extraction, although its incremental value over conventional imaging for this purpose has not been established in immature teeth. Contemporary trauma guidelines accordingly recommend CBCT, used selectively, for the assessment of root and crown–root fractures and lateral luxations, where it localizes the fracture and defines its extent and direction more reliably than periapical views. Because children are more susceptible to the stochastic effects of ionizing radiation, CBCT should not be a routine substitute for conventional radiography but should be justified case by case and acquired with the smallest field of view and lowest dose compatible with the diagnostic task, in accordance with the ALARA principle [37].

Importantly, radiographic appearance alone may not distinguish a normal developing apex from pathology in the immature tooth: the physiologically widened apical periodontal ligament space and the radiolucency of the developing apical papilla can mimic a periapical lesion. These findings must therefore be interpreted alongside the history, clinical examination, and pulp testing rather than in isolation. When uncertainty persists, monitoring over time is preferable to immediate intervention [38,39].

The optimal radiographic diagnosis and follow-up protocols for immature teeth undergoing apical barrier treatment nevertheless remain undefined: the selective indications for CBCT in this context are extrapolated from the general trauma literature rather than from studies of these teeth, and whether its additional three-dimensional information meaningfully alters treatment decisions or outcomes—enough to justify the added radiation burden in this radiosensitive group—has not been established. This issue represents a notable evidence gap.

#### 3.4.2. Isolation of the Immature, Frequently Traumatised Tooth

Effective rubber dam isolation is the standard of care for any non-surgical endodontic procedure, providing an aseptic field, protecting the airway, and improving access and visibility [40]. In the immature tooth, however, isolation is frequently complicated by factors peculiar to this group: the tooth may be incompletely erupted with a short clinical crown, and—because so many of these cases arise from trauma—the crown is often fractured, leaving insufficient sound structure to retain a clamp. Where the bulge of the tooth lies at or apical to the gingival crest, there are no undercuts to prevent the clamp sliding coronally [41].

Several adaptations address these difficulties. A pre-endodontic build-up of the broken-down crown with composite or glass-ionomer re-establishes four walls, facilitating both clamp retention and a sound access cavity. Alternative strategies include etching the enamel to bond small composite spots that grip the clamp, isolating several teeth together, anchoring the dam to adjacent teeth, or stabilizing it with fragments of dam material passed beneath the contact points instead of a clamp [41]. A specific and important caution applies to the recently traumatized tooth: where the periodontal ligament is injured, and the tooth is mobile, apically directed wedging forces from a clamp at the cemento-enamel junction can, in rare instances, displace or even avulse the tooth, so clamp placement must be gentle and, where possible, transferred to adjacent teeth [42]. Adequate isolation is not a trivial preliminary: contamination of a recently disinfected canal by oral fluids undermines the very disinfection on which apical barrier depends.

These isolation strategies, however, are supported only by case reports and expert opinion; the influence of the isolation method on the outcome of apical barrier in immature teeth has not been investigated clinically and remains a notable evidence gap.

#### 3.4.3. Access Cavity Preparation

In laboratory studies, the traditional access cavity has been considered more suitable than the conservative access cavity for treating immature permanent teeth with apical barriers, since the total porosity of the apical barrier with traditional access cavities was significantly lower than with conservative access cavities. Straight-line access in traditional cavities facilitates placement of the MTA barrier. In contrast, in conservative cavities, contact between the plug-transfer and condensation instruments and the chamber walls may restrict straight-line access to the apex, leading to a more porous fill than in traditional cavities [43].

No clinical study was found comparing the effect of access-cavity design on the outcomes of treating non-vital immature permanent teeth. These cases are often associated with traumatic crown fractures [44], such that a wide access is automatically secured along the fracture line.

#### 3.4.4. Working-Length Determination

Determining the working length is crucial to ensure accurate canal preparation, disinfection and filling. Periapical radiographs are used for this purpose without electronic apex locators in immature permanent teeth, since the absence of an apical constriction gives unreliable readings; canals larger than #80 were unreliable to measure because of the potential influence of periapical tissue fluids on the electronic apex locator reading [45,46]. Periapical radiographs are therefore preferred for this purpose [47].

Re-confirmation of the working length before the barrier formation visit is recommended when a long period elapses between treatment visits—particularly in infected cases, owing to the possibility of external resorption—or if the procedure is taken over by a different clinician, or if circumstances require modification of the original working length (such as a new traumatic injury between visits), or if the patient reports pain at a point shorter than the previous length, or in the event of loss or replacement of the coronal restoration between appointments, which may alter the length and the previous reference point [47].

The use of electronic apex locators in determining the working length and maintaining it during the preparation of closed-apex canals has helped to reduce post-operative endodontic pain, owing to a reduction in the inadvertent irritation of the periapical tissues; endomotors with an integrated apex locator and an apical auto-reverse function limit apical extrusion of debris and the consequent release of inflammatory mediators in the periapical tissues, and have been associated with less post-operative discomfort and lower analgesic consumption [48].

However, it remains unknown whether the more recent generations of electronic apex locators can be used with the large-sized hand and rotary files required for working-length determination and minimal mechanical preparation in immature permanent teeth, and how this would compare with the conventional radiographic method in terms of reducing post-operative pain following apical barrier treatment.

#### 3.4.5. Minimal Mechanical Preparation of the Immature Canal to Receive an Apical Barrier

Mechanical preparation of the root canals in immature permanent teeth is a controversial subject. Many researchers agree that conventional mechanical preparation may weaken the thin walls of immature teeth, increasing the risk of future root fracture; chemical cleaning alone using irrigants such as sodium hypochlorite is therefore preferred, primarily to reduce structural damage and preserve the remaining radicular dentine, while also avoiding unnecessary injury to any surviving cells of the apical papilla. A distinction should nonetheless be drawn between the two treatment aims: continued root development is a primary, intended objective of regenerative endodontic procedures, whereas apical barrier treatment is undertaken after confirmation of pulp necrosis and is directed chiefly at infection control and structural preservation. Even so, radiographic root lengthening and dentinal-wall thickening have been reported after apical barrier treatment in some immature teeth, suggesting that residual apical-papilla cells may retain regenerative potential; this outcome, however, is inconsistent and unpredictable rather than a reliable goal of the technique [49].

From another perspective, minimal mechanical preparation may be necessary to improve removal of the smear layer and necrotic pulp remnants and to eliminate the reverse taper established in the coronal and middle thirds, ensuring that irrigants and plug-formation instruments reach the apical third—provided this is done with great care and without additional weakening of the root walls. Modern techniques such as the XP-endo Finisher and Gentlefile files have provided safer alternatives, allowing access to complex areas within the canal while removing a relatively small amount of dentine compared with conventional files, as shown by laboratory studies using micro-computed tomography (micro-CT). The current principle can be said to rest on balancing preservation of tooth structure with adequate canal debridement, with a tendency to minimise or avoid excessive mechanical preparation in immature permanent teeth except when strictly necessary [6,8,10,50].

#### 3.4.6. Irrigants Used in Apical Barrier Treatment

Sodium hypochlorite is considered the gold standard among irrigants; it is an excellent solvent that dissolves living and necrotic organic pulp tissue and has effective activity against endodontic micro-organisms and biofilms [51]. There is insufficient research on the optimal time, volume, and concentration for treating non-vital immature permanent teeth.

The optimal sodium hypochlorite concentration for immature teeth has not been established. Although higher concentrations, such as 5.25%, have greater antibacterial and tissue-dissolving activity, this advantage must be weighed against the increased risk of apical extrusion and cytotoxic injury in the open-apex tooth, where the natural apical constriction is absent. Reported success rates do not resolve this question: high success has been described both with 5.25% hypochlorite followed by a calcium silicate barrier (84–92% depending on the cement [47]) and with a lower concentration of 1.3% hypochlorite followed by Q-Mix, both ultrasonically activated, ahead of MTA or Biodentine (100% at one year [6]). These figures derive from studies that differ in irrigant concentration, activation system, lesion characteristics, barrier material, and follow-up duration, and cannot be attributed to concentration alone. Rather than favoring a particular concentration, the evidence points to the importance of controlled, low-pressure delivery using side-vented needles, careful control of the working distance short of the apex, and selection of concentration according to the individual case.

There are some variations in the irrigation methods for non-vital immature permanent teeth during apexification, but these did not affect the overall outcomes or survival rates; a systematic review of apexification studies found that all relied on sodium hypochlorite as the principal irrigant, with EDTA and, less commonly, chlorhexidine used as adjuncts [10].

Incorporating EDTA into the irrigation regimens of non-vital immature canals has numerous advantages; it chelates calcium ions within the mineral dentine crystals, thereby limiting surface demineralisation and exposing the organic dentine matrix. As a result, growth factors stored within the dentine matrix—such as TGF-β, BMP, and VEGF—are released. The release of these factors provides biological signals that can stimulate the migration, proliferation, and differentiation of stem cells and support angiogenesis and mineralised-tissue formation [49,52,53]. These regenerative effects are central to the rationale of regenerative endodontic procedures. In apical barrier treatment of the necrotic immature tooth, the principal purpose of EDTA is instead smear-layer removal and conditioning of the dentine surface; any such bioactive effects are a secondary and less predictable consideration once pulp necrosis has been confirmed, although the residual regenerative potential of surviving apical-papilla cells may occasionally contribute to the continued apical development reported in some treated teeth [49].

Furthermore, by removing the smear layer, EDTA increases the penetration of calcium silicate particles into dentinal tubules, thereby enhancing the stability of the apical barrier and its push-out bond strength [54].

Regular replacement of the irrigant and the use of a large volume are essential to ensure maximal antibacterial efficacy [55]; nevertheless, there was variability among apical barrier studies regarding the volumes of irrigant used and the protocols employed in disinfecting immature canals [10].

#### 3.4.7. Irrigant Activation in Non-Vital Immature Permanent Teeth

Irrigant activation is of particular importance in non-vital immature permanent teeth, as it helps to improve the irrigant’s ability to remove necrotic tissue remnants, the smear layer and the bacterial load from areas inaccessible to conventional instruments. Activation of irrigants by various means (such as passive ultrasonic irrigation or modern instruments such as the XP-Endo Finisher or lasers) shows greater efficacy in increasing the penetration of solutions and enhancing their contact with the entire wall of the immature canal, thereby improving biofilm removal [56,57]. Achieving effective cleaning in these teeth raises the chances of success of endodontic treatment and reduces the need for additional mechanical preparation that may weaken the already weak root structure [58]. Nevertheless, no clinical studies have compared short- and long-term outcomes with and without activated irrigation.

The use of the XP-Endo Finisher in irrigant activation is an advanced and effective step for improving the cleaning of root canals in immature permanent teeth [59]. An in-vitro study showed that this file removes intracanal dressings from immature canals more effectively than conventional needle irrigation and ultrasonic activation [56]. Likewise, another in-vitro study showed that activating EDTA solution with the XP-Endo Finisher increases the release of transforming growth factor beta-1 (TGF-β1) from dentine, enhancing the biological dimension in conservative revitalisation protocols, especially with reduced EDTA irrigation time to avoid adverse effects on the thin dentine of immature teeth [60].

Laser-activated irrigation has been investigated as an advanced method for enhancing disinfection, specifically in immature permanent teeth. Photon-induced photoacoustic streaming (PIPS), which employs an Er: YAG laser to generate cavitation and photoacoustic shock waves, and the more recent shock wave-enhanced emission photoacoustic streaming (SWEEPS), produce a three-dimensional streaming of the irrigant that may improve its penetration and contact with the canal walls without the need for additional mechanical preparation—an attractive property in the thin-walled immature canal. In simulated immature-tooth models, however, laser-activated protocols have shown variable superiority over passive ultrasonic and sonic activation: studies have reported comparable or only marginally better removal of intracanal medicaments and dissolution of periapical organic tissue, with no consistent advantage of one laser modality over the other [61,62]. The evidence, therefore, suggests that while laser activation is a promising adjunct for disinfecting the immature canal, its benefit over established activation methods in this specific group remains to be clearly demonstrated.

Finally, bearing in mind that most studies did not test different disinfection methods for non-vital immature canals in clinical studies, success rates were generally high; nevertheless, the cellular and bacterial status of immature permanent teeth after treatment remains imprecisely known, as none of the studies reported performing bacterial sampling of immature canals as a standard method for verifying the efficacy of disinfection before the filling stage [10]. This evidence gap extends to the clinical role of irrigant activation in this group: Although the laboratory data summarised above are encouraging, there is a lack of clinical studies evaluating whether the choice of activation method influences clinically meaningful outcomes such as post-operative pain or the radiographic healing of periapical lesions in non-vital immature permanent teeth treated with apical barriers.

#### 3.4.8. Intracanal Dressings

Intracanal dressings are commonly used in apical plug protocols to reduce the bacterial load, control exudate, stimulate healing, and prepare the canal environment before plug placement. However, their necessity, optimal material, and duration remain unresolved. Calcium hydroxide is the most common dressing and has proven effective in forming the apical barrier over an average period of 3–12 months; it is also antibacterial and well tolerated by the periapical tissues and the stem cells of the apical papilla, but prolonged use may weaken the dentine walls and increase the risk of root fractures, warranting caution [63,64].

Calcium hydroxide dressing is also used as a preliminary step prior to placement of the apical plug, aiming to disinfect the canal and achieve its complete dryness in preparation for placement of the bioceramic barrier. A recent retrospective study on immature permanent teeth demonstrated that a staged protocol employing calcium hydroxide dressing for a period ranging from one to three months periodically replaced until complete canal dryness was achieved followed by a bioceramic putty apical plug, yields favorable outcomes; the strict success rate (complete radiographic resolution of the lesion) reached 83.1%, with a statistically significant reduction in the mean periapical lesion area at the final follow-up [65].

Triple antibiotic paste (metronidazole, ciprofloxacin, minocycline) has proven highly effective in disinfecting the root canals of non-vital immature teeth, showing the ability to eliminate bacteria resistant to calcium hydroxide, making it a promising option, especially in cases with a high bacterial load [64]. Its use as a short-term dressing (two to four weeks) before apical closure or regenerative procedures helps to obtain a clean canal environment suitable for healing [66].

The endodontic literature mentions the use of corticosteroid dressing (Ledermix paste) as an adjunctive dressing for managing severe luxation trauma cases by inhibiting root resorption, with a positive effect on treatment outcomes [67].

In addition to its role in disinfection, the intracanal dressing clinically is used as a preparatory step before placing the apical barrier (with materials such as MTA or bioceramic materials), as it helps to reduce bleeding and exudate and ensures the canal is dry and prepared for tight placement of the barrier, which improves the apical seal and long-term treatment success [47,68].

In a previous study [6], no calcium hydroxide dressing was used in the management of non-vital immature permanent molars; instead, Q-Mix irrigant was used to irrigate the immature canals and was activated in a single-visit procedure immediately before placing the apical barrier, and this method achieved high clinical and radiographic success. The optimal type, duration, and necessity of intracanal dressing for non-vital immature permanent teeth, and its appropriate use in apical plug treatment, remain unclear. These questions require future well-designed randomized controlled clinical trials.

A new intracanal dressing composed of calcium silicate (calcium silicate–based intracanal medicaments) has recently appeared, such as Bio-C Temp, which has shown promising properties, including high biocompatibility, the ability to stimulate cells and release growth factors from dentine, and the potential to support healing processes. Despite these properties, the current evidence remains mostly laboratory-based or experimental, with a clear lack of long-term clinical studies supporting its use within apical barrier protocols [69].

#### 3.4.9. Drying and Moisture Control of the Immature Canal Before Apical Barrier Placement

Before the apical plug is condensed, the canal should be freed of excess residual irrigant, blood and periapical exudate, since surplus fluid interferes with the adaptation and condensation of the material and may promote its extrusion through the open apex [70]. In the wide, open-apex canal this is more demanding than in the mature tooth, as standard paper points may not adapt to the apical third and periapical fluid can seep coronally through the patent foramen; drying is therefore usually achieved with large-diameter paper points while ensuring the absence of active bleeding before placement [71]. A distinction specific to these materials is that hydraulic calcium silicate cements set through a hydration reaction and are stable in a wet environment—indeed, ambient moisture is drawn from the dentine and periapical fluid to complete their setting—so that, unlike conventional gutta-percha and sealer, an excessively desic-cated field is neither necessary nor advantageous, and the clinical objective is the con-trol of excess moisture rather than complete dehydration of the dentine [72,73].

The influence of the residual moisture state on the material–dentine interface is, however, neither simple nor fully resolved. Laboratory studies have reported conflict-ing optima: for a bioceramic sealer, a dry field produced significantly higher push-out bond strength than wet or half-dry conditions, with no significant difference between the moist and dry states [74], whereas for another bioceramic sealer complete desiccation yielded the lowest push-out strength and paper-point drying to a controlled-moist state performed best [75]. Blood contamination of the apical field has likewise been shown to alter push-out bond strength in a material-dependent manner among MTA, Biodentine and TotalFill [76]. Taken together, these findings indicate that the moisture condition measurably affects adhesion and sealing, that the optimum differs between cements, and critically that all of this evidence derives from in vitro models of mature or simulated roots; the optimal moisture protocol for the immature canal specifically, and its influence on clinical outcomes, has not been established.

#### 3.4.10. Apical Barrier Formation Techniques

The formation of the apical barrier refers to the appropriate delivery and condensation of the material into the apical third of the canal to create an artificial apical barrier, after preparing and drying the canal [77]. After mixing, the material is placed at the canal orifice. A manual vertical plugger is then used to progressively compact and adapt it to the required thickness within the apical third. A plugger diameter of approximately 50% of the apical diameter has been suggested to allow effective condensation without the excessive force that may displace the material or damage the canal walls [78], although this figure derives from technique description rather than comparative clinical testing.

Several other specialised, non-conventional methods have been described in the literature, including the following:

**Material-Delivery Techniques**

**Lentulo spiral.** An old technique that delivers and distributes the material directly into the immature canal by loading it onto the lentulo spiral [79].

**Amalgam carriers.** The material is introduced into the orifice of the immature canal using an amalgam carrier and packed into the apical third using manual vertical hand pluggers [80], large gutta-percha cones [81], large paper points [82] or large hand files [71].

**Dedicated carriers such as the Micro-Apical Placement (MAP) System.** The MAP System allows precise delivery of the barrier material to the apical third via a fine, flexible, guided metal cannula, reducing the likelihood of material packing during condensation from the orifice towards the apex without the need for excessive canal enlargement; the material is then injected into the apical third and condensed using manual vertical pluggers [6,77,83].

**Thermafil carriers.** This technique is designed for curved canals with apices larger than #40. It involves removing the gutta-percha sheath from the flexible plastic carrier of the Thermafil warm-obturation system. Once a carrier appropriate to the canal has been selected (reaching 1 mm short of the radiographic apex), it is loaded with small, compacted pellets of calcium silicate cement, which are delivered to the apex and condensed with the same carrier to produce a 3 mm-thick plug [84].

**Modified cannula.** A patented Syrian device available in multiple sizes to suit immature teeth, consisting of a flexible plastic tip that can carry the barrier material and a flexible, closed-tip metal plunger that pushes the material once it reaches the appropriate location and condenses it [77].

**Barrier Condensation Techniques**

**Condensation using the XP-Endo Shaper.** A portion of the barrier material is placed using the dedicated carrier, then the XP-Endo Shaper is introduced into the canal to 1 mm short of the working length at 800 rpm in a counter-clockwise direction; on reaching the working length it is gently withdrawn while the file continues to rotate, ensuring homogeneous distribution of the material, and this process is repeated three times [43,78].

**Condensation using indirect ultrasonic activation of the manual vertical plugger.** The barrier material is placed in the canal in three increments (2 mm, then 2 mm, then 1 mm); during condensation of the increments with the manual vertical plugger, a fine ultrasonic tip (such as CPR-1) is activated in direct contact with the plugger—without the plugger touching the dentinal walls—while compacting the material into the apical third. The ultrasonic tip may be used at low, medium or high power [43,78,85], provided the duration of ultrasonic activation does not exceed 8 s, to avoid increased porosity or reduced microhardness in certain materials such as MTA [86].

**Prefabricated BioRoot inlay.** Described in two case reports, this principle is based on taking a light-body impression of the immature canal, then embedding this impression in heavy body to leave an imprint of the canal in the heavy body. After removing the light-body impression, the canal imprint in the heavy body is filled with barrier material; once set, the heavy body is trimmed to obtain a custom piece of barrier material ready for direct placement in the canal with an appropriate sealer [87,88].

Building on this concept, an in vitro study using three-dimensionally printed root canal models of immature permanent teeth developed the idea further by incorporating a fibre post into a BioCeramic putty Bio-Root inlay: the post, bonded to the set putty, serves as a handle by which the prefabricated Bio-Root inlay can be carried, tried into the canal and withdrawn, after which a sealer is applied and the unit is reseated, with the same fibre post subsequently used to support the definitive coronal–radicular restoration. That study compared different cementation systems for bonding the fibre post to the BioCeramic putty and reported the highest pull-out bond strength with a total-etch dual-cure resin cement. On this basis, it proposed the post-retained prefabricated-plug approach as a means of combining apical sealing with internal reinforcement of the thin-walled immature root [89].

A contradiction was found in the literature regarding the quality of apical barriers condensed by different techniques: barriers formed by delivering the material directly to the apical third and condensing it produced a better seal than those formed with an amalgam carrier that pushes the material into the coronal third to be subsequently packed into the apical third [77]. Condensation of MTA increments combined with indirect ultrasonic activation, or use of the XP-Endo Shaper to distribute the barrier material, increased the proportion of voids and porosity in apical barriers performed on extracted teeth [43]; conversely, indirect ultrasonic activation at high or medium power was found to reduce the marginal leakage of MTA barriers [85]. It was also found that both the XP-Endo Shaper and indirect ultrasonic activation reduced the external voids of barriers formed from other calcium silicate cements (Biodentine, NuSmile NeoPUTTY and Well-Root PT) performed on three-dimensional printed immature-canal analogues [78]. The optimal method for forming the apical barrier (including delivery and condensation) remains unclear for each material, and further research is required.

Previous studies have described the possibility of using the apical matrix placement technique before condensing the apical barrier; this matrix is usually made from collagen sponge, demineralised freeze-dried bone powder or platelet-rich fibrin, to support barrier formation in very wide canals or those with divergent apical walls before introducing the apical barrier material [90,91,92]. This technique helps to reduce extrusion of the barrier material beyond the apex, and there is scant information for short-term follow-up periods that it does not affect radiographic healing [91]. However, there are no systematic reviews or long-term studies comparing the use of this technique with its non-use and with extrusion of the barrier material beyond the apex. The root filling should ideally extend only to the level of the apical foramen. Where materials are extruded beyond the apex, whether the apical matrix or the filling material, long-term follow-up is needed to assess the effect on the healing of periapical lesions and on periapical bone repair.

The apical barrier technique may itself affect post-operative pain as a short-term outcome determinant: A single gutta-percha cone with a bioceramic sealer has been reported to cause less immediate post-operative pain than a bioceramic-putty apical barrier in necrotic immature incisors [59].

#### 3.4.11. Thickness of the Formed Apical Barrier

The optimal thickness of the apical barrier is one of the decisive factors in ensuring the seal and strength of teeth with open apices. Forming an apical barrier 3–5 mm thick achieves an adequate seal and reduces push-out of the apical barrier in laboratory studies [93]. Increasing the thickness of apical barriers from 1 mm to 3 mm reduces marginal leakage [94], whereas the seal of Biodentine and bioceramic putty barriers may not be affected by barrier thickness provided it is not less than 3 mm [95]. As the thickness of MTA or Biodentine barriers increases towards a complete fill, the fracture resistance of the immature permanent tooth decreases [96,97], whereas a barrier of 3–6 mm thickness reduces the risk of fracture [96]. An apical barrier thickness of less than 4 mm increases the likelihood of clinical failure one and a half times because of apical leakage [47]. On the basis of this predominantly laboratory evidence, together with limited clinical data [47], an apical plug of approximately 4–5 mm is frequently proposed as a pragmatic target, irrespective of the calcium silicate cement used [47,98]; this threshold has not, however, been validated in controlled clinical trials in immature teeth.

Within the literature reviewed for this narrative synthesis, no clinical studies were identified that directly compared outcomes. No clinical evidence was found to favor a complete fill or a 4–5 mm barrier for immature permanent teeth based on their degree of development. For immature permanent teeth with extensive breakdown requiring a coronal–radicular restoration, it is preferable to keep at least the coronal half of the immature canal empty to support the broken-down coronal structures with a coronal–radicular restoration such as fiber posts [99].

#### 3.4.12. Materials Used as Apical Barriers

The materials most commonly used as apical barriers belong to a single family: hydraulic calcium silicate–based cements, also termed bioceramic cements. They set in the presence of moisture, are biocompatible and bioactive, and form hydroxyapatite at the material–dentine interface [100,101]. Rather than representing distinct classes, the various commercial products differ chiefly in two respects: their presentation (operator-mixed powder–liquid or powder–gel systems versus pre-mixed, ready-to-use formulations) and their consistency (putty-type barrier materials versus flowable sealers) [102,103]. Table 1 compares the principal calcium silicate materials used for apical barrier placement.

Mineral trioxide aggregate (MTA) is considered the first calcium silicate cement to be applied in the field of root canal system treatment and is the most studied bioactive material to date. MTA was developed starting from Portland cement and possessed good biocompatibility and a high capacity for apical sealing. The inherent limitations of MTA include its long setting time, high cost and potential to cause tooth discolouration [101].

Further bioactive materials were developed in the late 2000s and early 2010s, possessing biological properties similar to MTA, such as antibacterial activity, low cytotoxicity and a moderate inflammatory response [104,105]. These modern materials—such as Biodentine, the bioceramic EndoSequence Root Repair Material (ERRM), BioAggregate and calcium-enriched mixture (CEM)—have been used extensively in clinical practice [106].

Within this family, the pre-mixed, ready-to-use formulations are frequently marketed as “bioceramic” putties, pastes, and sealers. They share the same calcium silicate basis as MTA and Biodentine but are supplied pre-mixed, which improves handling and reduces the risk of discolouration; the term therefore denotes a presentation format rather than a separate material class [107].

BioAggregate, CEM, TheraCal LC, EndoSequence Fast-Set Putty and BC Sealer HiFlow are mentioned as modern calcium silicate materials used as apical barriers; the use of calcium phosphate as an apical barrier has also been recorded [108,109].

Comparable clinical results with high success rates were found for both MTA and Biodentine as apical barriers when treating non-vital immature permanent molars with medium-sized periapical lesions in children; post-operative pain one day after treatment was lower in the MTA barrier group, whereas treatment time was shorter in the Biodentine group, and some cases in the Biodentine group showed a calcified tissue barrier apical to the barrier that did not appear in the MTA group [6].

A similar clinical study found that Biodentine was better in short-term outcomes (3 months) as an apical barrier for the healing of periapical lesions compared with MTA and calcium phosphate cement, whereas calcium phosphate cement was best in terms of radiographic healing in the long-term outcomes (9 months); a criticism of this study is its failure to specify whether the teeth involved were anterior or posterior [109].

A recent similar clinical study including maxillary central incisors found that both MTA and BioCeramic Putty were comparable in terms of resolution of clinical symptoms after treatment, whereas BioCeramic Putty outperformed MTA in terms of radiographic healing of periapical lesions in terms of the bone density restored at the lesion site [15].

A retrospective study was conducted on children under 16 years of age whose non-vital immature permanent incisors were treated, and found that the success rate reached 84% for MTA, 88% for Biodentine and 92% for TotalFill Putty after 12 months of apical barrier procedures. All materials achieved excellent clinical performance in apical barrier procedures; the use of MTA was associated with the highest rate of crown discolouration, whereas Biodentine was most associated with extrusion beyond the apex [47].

The individual comparisons summarised above should be read with caution: each derives from a single small clinical, retrospective, or in vitro study, and none has been replicated. Despite the breadth of materials now available as apical barriers, the comparative clinical evidence in immature permanent teeth remains limited and heterogeneous. Direct comparisons are derived largely from in vitro models, short-term clinical studies (3–12 months) or retrospective data, and the reported success rates of the principal materials are closely clustered and difficult to separate; no long-term randomised clinical trials have directly compared the newer pre-mixed bioceramic putties with MTA and Biodentine in this group. This uncertainty is compounded by inconsistent outcome criteria and uncontrolled confounders (such as tooth location, lesion size and follow-up duration) which limit comparison across studies.

#### 3.4.13. Management of the Residual Canal Space and the Definitive Restoration

A caution regarding irrigation of the residual canal after barrier placement. Where the apical barrier is placed and the remainder of the canal is irrigated in the same or a subsequent visit (before obturation or restoration) the irrigant brought into contact with the already-set cement merits consideration. An in vitro study on set ProRoot MTA demonstrated that contact with 2% chlorhexidine significantly increased marginal leakage and impaired the sealing ability of the hardened cement [110]. Although this evidence derives from a furcal-perforation model rather than from apical barriers specifically, the underlying principle is directly transferable: once a calcium silicate apical barrier has set, flushing the residual canal with chlorhexidine may compromise the very apical seal on which the success of apical barrier depends, and an inert final rinse such as saline is therefore preferable in contact with the set barrier. The influence of post-placement irrigation on the seal of calcium silicate apical barriers has not been investigated directly, and represents a further procedural variable warranting dedicated study.

A well-sealed coronal restoration is no less important than the apical seal in the management of the immature permanent tooth, both in preventing coronal leakage—a well-recognized cause of endodontic treatment failure—and in restoring the missing portion of the immature tooth’s coronal walls [111]. The thin, divergent dentinal walls and the weak pre-cervical dentine leave these teeth highly susceptible to cervical fracture, which is among the principal causes of late tooth loss after apical barrier [10,68].

In most cases, where coronal tooth structure is largely intact, the canal space coronal to the apical barrier is obturated conventionally with gutta-percha and a suitable sealer, and the access cavity is then restored with a bonded resin composite, which both seals the canal coronally and restores function and aesthetics. A post is not routinely required in such teeth, and is reserved for situations in which extensive loss of coronal tissue demands additional retention and reinforcement.

Two restorative principles follow from this. First, as mentioned previously, sufficient canal space should be preserved coronal to the apical barrier to allow placement of an intra-radicular fibre post when the coronal tissue is extensively broken down; a complete fill that leaves no room for a post may be counter-productive in such cases [112,113]. Laboratory evidence indicates that restoring simulated immature teeth with a fibre post and composite resin after a calcium silicate apical barrier increases their fracture resistance, supporting the concept of internal reinforcement of the thin-walled root [112]. Second, because the most widely used barrier materials—particularly MTA—carry a recognised potential for crown discolouration, contact between the cement and the coronal dentine should be avoided, the pulp chamber cleaned of residual material, and consideration given to sealing the coronal dentine to mitigate aesthetic impairment in these often anterior, aesthetically critical teeth [114]. The timing and choice of definitive restoration should therefore be planned from the outset, as an integral part of the treatment, rather than as an afterthought once apical closure has been confirmed.

Two evidence gaps follow. First, the irrigant most appropriate for cleansing the residual canal coronal to the set barrier before restoration is undefined. As noted earlier, the final rinse in contact with the set cement can affect its seal, yet the optimal choice has not been established for each combination of barrier material and restorative or reinforcement technique. Second, the evidence for internal reinforcement is largely confined to in vitro testing of conventional fibre posts. Alternative strategies—customised or short composite-resin posts, three-dimensionally printed root substructures, and bondable polyethylene-fibre systems (e.g., Ribbond)—remain poorly characterised in the laboratory and, especially, clinically. No studies link them to the long-term survival of apexified immature teeth. Both warrant standardised in vitro comparison and prospective clinical evaluation.

Beyond the immature tooth specifically, evidence from vital pulp therapy reinforces the broader principle that calcium silicate cements should not be evaluated independently of the definitive restorative protocol. In a three-year clinical case series, stable outcomes were reported when bioactive calcium silicate materials were integrated with immediate or staged adhesive restorations providing an effective coronal seal [115]. This observation constitutes indirect evidence, as it derives from vital mature posterior teeth rather than necrotic immature teeth; nonetheless, it is consistent with the concept (developed throughout this review) that material bioactivity and restorative integrity are complementary, rather than independent, components of a tooth-preservation strategy.

## 4. Factors Affecting the Outcomes of Apical Barrier Treatment

### 4.1. Effect of Operator Experience on Treatment Outcomes

Operator experience appears to be an important factor in the success of root canal treatment: observational studies have reported that higher technical competence during the various stages—isolation, access-cavity preparation, mechanical and chemical debridement, placement of intracanal dressings, and canal filling—is associated with higher healing rates [116,117,118]. Some research has indicated that patient satisfaction is higher when treatment is received from experienced specialists, underscoring the importance of clinical skill in improving the treatment experience [117,119,120].

In a single retrospective study, the treatment failure rate was reported to be almost twice as high when apical closure or filling was performed by less experienced clinicians, and treatments carried out over more than one appointment or by different clinicians were more prone to failure; the authors attributed this to a lack of continuity of care and variation in skill level, particularly where the temporary restoration was lost or contaminated between visits [47]. As these findings derive from retrospective, observational data, they should be regarded as a plausible hypothesis rather than as established causal evidence.

Most systematic studies that evaluated apical barrier treatment outcomes were conducted in specialised centres by highly experienced practitioners, which limits the generalisability of their findings to general practice, where the vast majority of endodontic treatments worldwide are performed in general clinics—often without the aid of the surgical microscope or specialist instruments. This may explain the discrepancy in success rates between academic studies and real-world clinical practice [2,10].

### 4.2. Effect of Child Cooperation

Treatment may be further complicated by high levels of dental fear and anxiety in children, particularly with regard to local anaesthesia, which may cause problems in patient cooperation and increase the stress of patients and parents [121,122]. Similarly, each stage of endodontic treatment may represent a new and unfamiliar experience for the patient [123]. It is therefore important to document the challenges of patient cooperation, as they may also affect the quality of endodontic treatment [45], in addition to the fact that children are susceptible to dental fatigue during the long treatment periods associated with traumatic dental injuries [124].

A retrospective study confirmed that child cooperation was among the factors most influencing the success of endodontic treatments performed in children [45]. The child’s behaviour during treatment, the difficulty of direct visualisation of the immature permanent tooth, and the precision of placing the material in the ideal location may also increase the risk of tooth discolouration [10].

Moreover, uncooperative children required a longer overall duration of the apical barrier procedure, irrespective of the apical barrier method employed [59].

### 4.3. Effect of the Severity of the Traumatic Injury or the Cause of Pulp Necrosis as a Prognostic Factor

Traumatic dental injuries increase the risk of early loss of immature permanent teeth; evidence indicates that luxation injuries adversely affect the prognosis of fractured teeth. Isolated crown fractures show high rates of pulp survival, whereas the presence of a concomitant injury to the supporting tissues leads to a marked increase in the likelihood of pulp necrosis owing to disruption of the neurovascular bundle at the apex and impairment of the pulpal blood supply. These injuries are also associated with an increased likelihood of subsequent complications such as inflammatory root resorption and periapical inflammation, which may negatively affect long-term treatment success. It is believed that injury to the periapical tissues and the periodontal ligament weakens the capacity for natural biological repair and affects the revascularisation process, thereby reducing the chances of healing and worsening the clinical prognosis of the injured teeth [125,126,127]. Nevertheless, a meta-analysis showed scope for bias, as the studies conducted on non-vital immature permanent teeth include teeth that became non-vital because of traumatic injuries and teeth that became non-vital for other reasons, without clarifying the effect of this factor on long-term treatment outcomes; this analysis nonetheless reported high survival rates for all treated non-vital immature permanent teeth included overall [2]. Another systematic review, including studies with long follow-up periods, indicated that the severity of the dental trauma itself—such as complete avulsion—may affect the long-term prognosis of the teeth more than the treatment technique used in managing the immature permanent tooth [10].

### 4.4. Effect of the Presence of Root Resorption

Immature permanent teeth are rapidly susceptible to root resorption because of the wide dentinal tubules that allow bacterial penetration [128]. Inadequately disinfected canals are more prone to inflammation or external root resorption, particularly those whose canals are associated with long periods between pulp-extirpation and filling appointments, which may lead to a change in apical anatomy and consequently a change in the working length. A retrospective study indicated that the presence of root resorption before the start of treatment is associated with a 3.5-fold increase in the likelihood of treatment failure [47].

### 4.5. Effect of the Level of the Root Filling on Success

The level of overfilling, underfilling or adequate filling when using MTA barriers in apical barrier treatments did not affect the overall clinical and radiographic success [67], and there were no notable differences in periapical lesion healing between non-vital immature permanent teeth filled with MTA barriers to an over-extended level or within the ideal limits [129]. This may be explained partly by the good biocompatibility of MTA [130] and by the ability of MTA to induce the deposition of mineralised tissue at the site of application [131].

Extrusion of the bioceramic material beyond the apex was not associated with increased postoperative pain in the treatment of immature permanent teeth [6].

There are no recent studies that have precisely evaluated the outcomes of over- or under-filling using the more modern calcium silicate cements such as Biodentine or BioCeramic, and the long-term effects of extrusion of calcium silicate cements remain imprecisely evaluated to date [10].

### 4.6. Effect of the Presence of Periapical Lesions on Treatment Success

The absence of a pre-operative apical radiographic lesion significantly improves the outcomes of endodontic treatment in teeth with closed apices [117], and the size of the apical lesion negatively affects the outcomes of treatment with the single-cone technique with bioceramic sealer in teeth with closed apices [132]. For non-vital immature permanent teeth treated with MTA barriers, the presence of a large lesion (grade 3–5 on the PAI score) increases the failure rate two- to four-fold [133], whereas the presence or absence of apical lesions in immature permanent teeth treated by apical barrier using bioceramic putty was found not to affect the success rate [134].

Meanwhile, another retrospective study identified four independent factors associated with delayed complete radiographic healing of periapical lesions: Anterior tooth location, considered more prone to failure; a larger initial lesion area (≥50 mm^2^), which increases bacterial virulence and is therefore more likely to lead to failure; a wider apical foramen diameter (≥1 mm), which hinders the achievement of a tight seal and thus increases the likelihood of failure; and the presence of a sinus tract, regarded as an epithelialized communication between the periapical tissues and the adjacent tissues that complicates infection control and thereby raises the probability of failure [65].

These findings are limited by the factors discussed in this section. Almost all reported relationships between operator experience, continuity of care, trauma severity, resorption, filling level, or lesion size and the eventual outcome derive from retrospective or observational data, in which these variables are not independent of one another. Referral patterns, case complexity, lesion severity, the stage of root maturity at presentation, patient behavior and cooperation, and continuity of care may all covary with both the exposure and the outcome, and none of the available studies was designed to adjust for them. The associations reported here should therefore be interpreted as hypothesis-generating rather than causal, and residual confounding cannot be excluded. Prospective studies that explicitly record these variables and stratify or adjust for case complexity and operator level would be required to establish which of these factors genuinely influences outcome.

## 5. Assessment of the Outcomes of Apical Barrier Endodontic Treatment in Immature Permanent Teeth, Follow-Up Duration and Prognosis

Apical barrier treatment had generally better clinical and radiographic success rates among all available endodontic treatments for non-vital immature permanent teeth [135].

Clinical success is defined as the functional retention of the teeth without any signs of self-reported pain, pain on percussion or palpation, pathological tooth mobility, abnormal periodontal probing depth, swelling or fistula formation. Radiographic success is defined as evidence of healing of periapical tissue inflammation (if present) and the absence of any pathological signs on radiographs, such as internal or external root resorption. These criteria are based on most of the recent guidelines and systematic reviews concerned with the long-term outcomes of apical barrier treatments [2,10,136].

These outcome domains should, however, be distinguished rather than combined. Healing refers to the resolution of periapical inflammation, judged radiographically and clinically. Success denotes the achievement of predefined clinical and radiographic criteria, as above, and is the strictest of these measures. Survival refers simply to the retention of the tooth in function, and a tooth may survive while exhibiting persistent periapical radiolucency, discolouration, structural compromise, or the need for retreatment. Retention is narrower still, denoting only that the tooth remains in the mouth, irrespective of its condition. Reported outcome rates are therefore not directly comparable across studies unless the underlying definition is specified: survival rates are consistently higher than success rates for the same cohort, and pooling them conflates categorically different outcomes [137,138,139,140].

Radiographic assessment is usually the principal (and often the only) objective method used to evaluate endodontic treatment outcomes. It is performed using intraoral radiographs according to a set of criteria for determining the status of the apex, and these criteria are considered the gold standard for measuring endodontic outcomes in immature permanent teeth [10].

The periapical index (PAI index) is considered the most widely used criterion in recent studies that evaluated the outcomes of treatment with apical barriers in terms of the presence or absence of periapical bone inflammation at the initial assessment [6,15,141]; it comprises five scores, with PAI 2 and PAI 3 designated as the cut-off between apical health and the presence of apical disease [142].

The most important radiographic outcomes recorded in studies concerned with apical barrier were healing of apical lesions, apical closure (formation of a calcified tissue barrier), the absence of radiographic signs of fracture, and the absence of signs of internal or external root canal resorption; high success rates were recorded, ranging between 81% and 100% [6,10,109,135,141,143]; this range reflects heterogeneity in outcome definitions as well as in case selection and follow-up duration, since the individual studies varied in whether they reported success, healing, or survival.

Opinions vary regarding the ability of apical barrier treatment to promote radiographic root growth in non-vital immature permanent teeth. Some studies confirmed that the use of MTA as an apical barrier did not contribute to an increase in root length [2,6,10], whereas Biodentine barriers showed a slight increase in the root in a limited number of cases [6]. Another study observed that the use of MTA as an apical barrier led to only slight increases in root length compared with revascularisation procedures after a one-year follow-up period [144], while some case reports indicated that the use of MTA or bioceramic putty as apical barriers led to an increase in root length of a distance equal to or exceeding 2 mm [145,146].

It is worth noting that post-operative pain was reported as a secondary short-term outcome variable of endodontic treatment of immature permanent teeth using apical barriers, and was often measured by its presence or absence or using the visual analogue scale (VAS) [59]; the most important clinical outcomes obtained were the absence of local signs of infection (fistula and abscess), the absence of symptoms, and a low incidence of root fractures [6,10,15].

Discolouration of immature permanent teeth treated with MTA apical barriers was considered a treatment complication owing to its effect on aesthetics [10]; however, where the clinical and radiographic signs of disease resolve, discolouration is not conventionally classified as a treatment failure [147]. This illustrates the distinction drawn above: such a tooth meets the criteria for healing and survival, yet remains aesthetically compromised, and outcome reporting that records only success or failure will not capture this.

The European Society of Endodontology recommends following up non-vital immature permanent teeth at 3, 6, 12, 18 and 24 months, then annually for five years [7,10]; nevertheless, recent studies have provided a shorter follow-up duration for the included samples, with only short-term outcomes ranging between nine months and three years [6,15,47,109,141]. Table 2 summarises, for each procedural step, what is reasonably supported, what remains uncertain, and the predominant type of underlying evidence. More robust studies are warranted to strengthen the evidence-based approach.

## 6. Equity, Training, and Global Implementation Considerations

**Figure 1:**
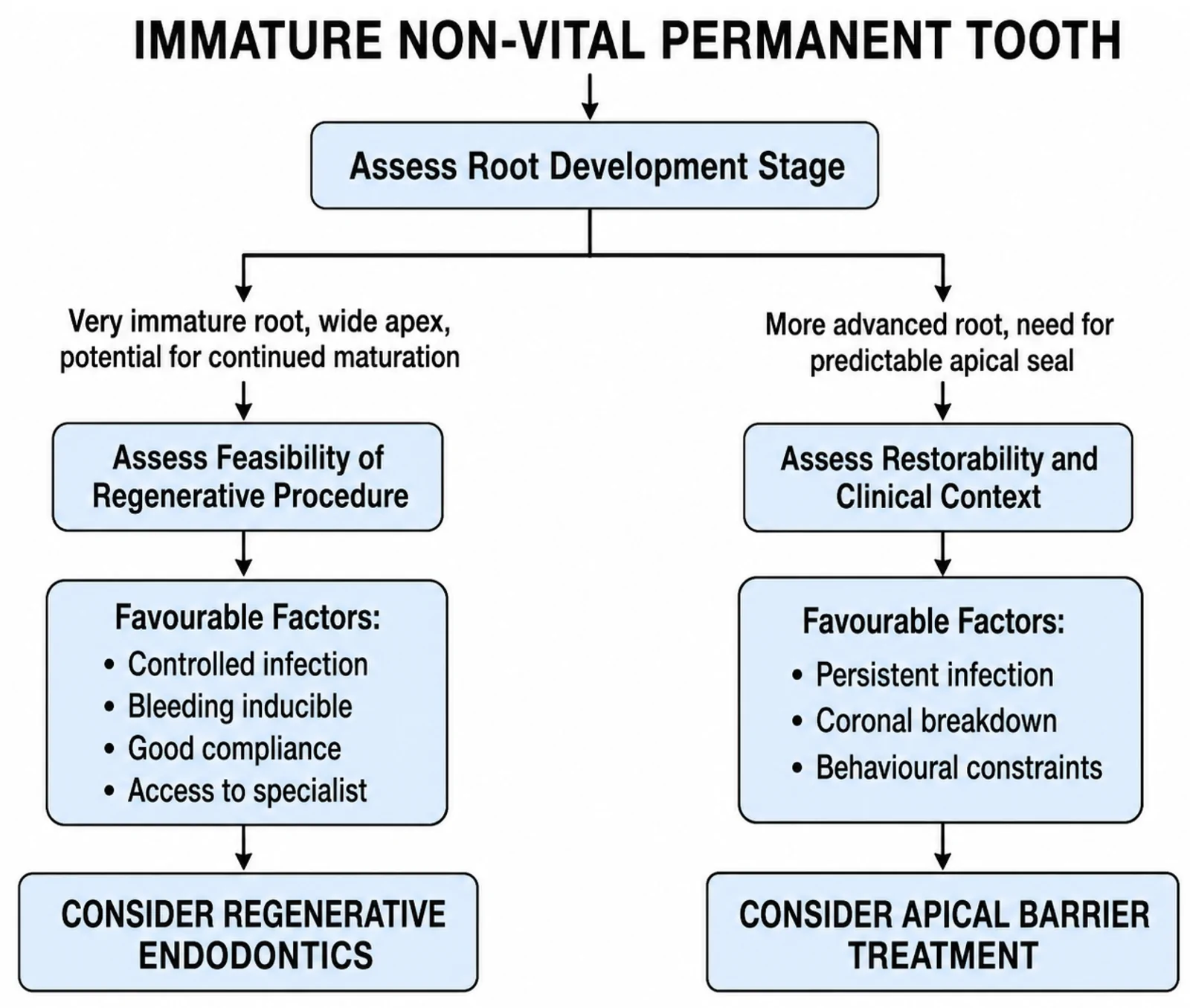
Decision Algorithm—Apical Barrier vs. Regenerative Endodontics.

Apical barrier therapy should not be viewed solely as a material-driven technique. In many health systems, the prognosis of immature permanent teeth is shaped by delayed trauma care, limited access to specialists, the cost of bioceramic materials, the availability of rubber dam isolation, radiographic resources, behavioral support, and the clinician’s training. Effective preservation of these teeth therefore requires not only selecting the appropriate calcium silicate material but also ensuring that children can access timely diagnosis, skilled endodontic care, definitive restoration, and long-term follow-up. Without these system-level conditions, technologically advanced procedures may widen rather than reduce disparities. Future research should therefore report not only healing and survival but also access barriers, treatment delays, operator training level, setting of care, cost, retreatment burden, and child-centered outcomes. Table 3 outlines the decision boundary between apical barrier and regenerative endodontics, and Figure 1 presents a clinical decision algorithm to guide this choice in practice, as the setting of care is valuable for pediatric dentists or endodontists.

## 7. Limitations of This Review

This manuscript is a narrative review rather than a systematic review; therefore, formal risk-of-bias assessment, meta-analysis, and quantitative grading of certainty were not conducted. The reviewed literature is heterogeneous with respect to case definition, root-development stage, tooth type, etiology of necrosis, lesion size, material selection, barrier placement technique, restoration type, follow-up interval, and outcome criteria. In addition, many procedural recommendations are derived from laboratory studies using simulated immature teeth, and their clinical relevance remains uncertain. These limitations should be considered when interpreting recommendations on irrigation, activation, barrier placement, moisture control, reinforcement, and material selection.

The interpretation of findings, as summarized in Table 4, is further constrained by the predominantly retrospective nature of clinical outcome data and the lack of studies that verify canal disinfection through bacterial sampling. Consequently, the strength of evidence for specific procedural recommendations varies substantially, and readers should distinguish between recommendations based on multiple clinical studies and those derived primarily from laboratory or extrapolated evidence.

## 8. Clinical Pearls for Apical Barrier Treatment

**Figure 2:**
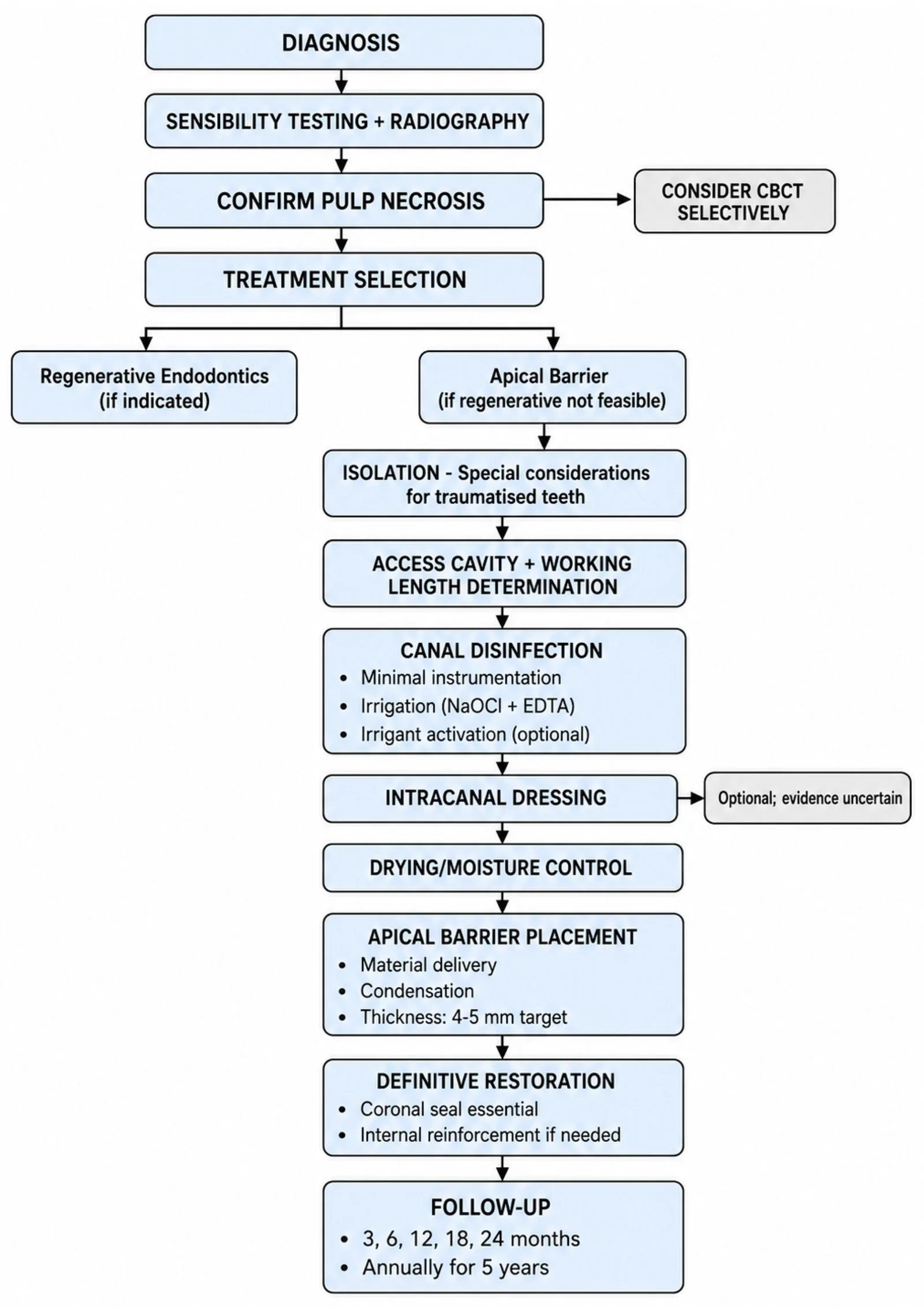
Clinical Workflow for Apical Barrier Treatment.

Based on the evidence synthesized in this review, the following clinical pearls may guide practitioners:

1. Diagnosis: Do not rely on a single sensibility test in immature teeth. Integrate history, clinical examination, and radiographic findings.
2. Isolation: Plan for difficult isolation in traumatised teeth. Pre-endodontic build-up or alternative clamp strategies may be required.
3. Working Length: Use periapical radiographs; electronic apex locators are unreliable in wide, open-apex canals.
4. Disinfection: Use controlled, low-pressure irrigation with side-vented needles. EDTA is recommended for smear-layer removal.
5. Barrier Placement: Target 4–5 mm thickness. Choose material based on clinical context and operator familiarity.
6. Restoration: Coronal seal is as important as apical seal. Plan the definitive restoration before barrier placement.
7. Follow-up: Monitor at 3, 6, 12, 18, and 24 months, then annually for 5 years. Figure 2 provides a visual summary of the clinical workflow for apical barrier treatment.

## 9. Conclusions and Future Directions

Apical barrier placement with hydraulic calcium silicate cements is a predictable, well-established treatment for non-vital immature permanent teeth, providing immediate apical closure, a short treatment duration, and high clinical and radiographic success across the principal materials (MTA, Biodentine, and pre-mixed bioceramic putties). Within this material class, observational evidence suggests as a hypothesis requiring prospective confirmation that the choice of material is less decisive for the outcome than the quality of disinfection, the adequacy and condensation of the barrier, the definitive coronal restoration, and the experience of the operator.

The pathway is nonetheless supported by evidence of uneven strength. The following priorities, ordered by clinical importance rather than by procedural sequence, represent the most pressing directions for future research:

1. Standardised outcome reporting. Heterogeneity in case definition, root-development staging, follow-up duration, and success criteria—and the frequent conflation of success, healing, survival, and retention—limits comparison across studies. A consensus framework for reporting apexification outcomes, alongside adequately powered randomised trials with long-term follow-up, is the single most important requirement.

2. Disinfection and its end-point. The microbiological status of the immature canal at the time of barrier placement is essentially unknown, as no study has verified disinfection by bacterial sampling. The optimal irrigant concentration, volume, and activation method for these wide, thin-walled canals remain undefined, as does the necessity, choice, and duration of intracanal dressing.

3. Barrier thickness, material comparison, and the operator effect. No long-term randomised trial has directly compared the newer pre-mixed bioceramic putties with MTA and Biodentine in immature teeth, and the optimal barrier thickness as a function of root-development stage has not been established clinically. Because retrospective data suggest that operator-related factors may influence outcome, future trials should record and adjust for operator experience so that any true material effect can be isolated.

4. The definitive restoration and internal reinforcement. Evidence for internal reinforcement is largely confined to in vitro testing of conventional fibre posts; alternative strategies remain poorly characterised, with no studies linking them to long-term survival. The optimal final irrigation regimen in contact with the set barrier, and the trade-off between apical seal and the canal space required for coronal–radicular reinforcement, are also undefined.

5. Diagnosis and the radiographic protocol. No validated reference standard exists for confirming pulp necrosis in the immature tooth, and the indications for CBCT in this context are extrapolated from the general trauma literature rather than established for these teeth.

Until such evidence is available, clinical decisions should rest on preserving tooth structure, achieving thorough disinfection, forming an adequate and well-condensed apical barrier of at least 4–5 mm, and providing a definitive coronal restoration—ideally delivered by an experienced operator.

Future studies should also incorporate child- and family-centred outcomes: pain experience, dental anxiety, number of visits, need for sedation or general anaesthesia, school absence, aesthetic satisfaction, quality of life, and caregiver burden. Because apical barrier therapy commonly follows traumatic injury in children and adolescents, biological success alone may not capture the full impact of treatment.

## Figures and Tables

**Table 1:** Comparison of Principal Calcium Silicate Materials for Apical Barrier Placement.

Material	Presentation/Setting Mechanism	Setting Time	Key Advantages	Key Limitations/Considerations	Discolouration Potential	Current Clinical Evidence Level
Mineral Trioxide Aggregate (MTA)	Powder–liquid (operator-mixed); moisture-dependent setting	~3–4 h	Most extensively studied; excellent biocompatibility and sealing; induces hard tissue deposition.	Long setting time; difficult handling; high cost; requires multiple visits for optimal set in some protocols.	High (well-documented crown greying)	Extensive (gold standard comparator in most trials)
Biodentine	Powder–liquid (capsule); mixed in triturator	~12 min	Faster setting than MTA; good handling; comparable biocompatibility; lower cost than some bioceramics.	Higher reported extrusion rate beyond apex; requires immediate coronal seal to prevent washout.	Moderate (less than MTA, but cases reported)	Growing (multiple RCTs and retrospective studies)
Pre-mixed Bioceramic Putties (e.g., EndoSequence BC, TotalFill)	Ready-to-use syringe (pre-mixed); sets in moisture	~4–24 h (depends on canal moisture)	Excellent handling; no mixing; no associated discolouration; good flowability and adaptability.	Higher cost; technique-sensitive; requires specific moisture control to set effectively; less long-term data.	Very Low/None	Emerging (promising short-term and retrospective data; lacks long-term RCTs vs. MTA)
Other Materials (BioAggregate, CEM Cement)	Powder–liquid/various	Variable (~2–4 h)				

**Table 2:** Summary of Supported Findings, Uncertainties, and Evidence Quality at Each Procedural Step.

Clinical Step	What is Reasonably Supported	What Remains Uncertain	Predominant Evidence Base	Clinical Certainty *
Diagnosis	Sensibility tests are unreliable in immature teeth; serial monitoring and integrating history/radiographs are critical.	Objective vitality thresholds and reference standards for confirming necrosis.	Clinical studies and guidelines	High
CBCT	Useful selectively for trauma, resorption, and fractures when conventional imaging is insufficient.	Whether CBCT changes treatment decisions/outcomes in apexification; incremental value over periapical views.	Guidelines and indirect clinical data	Moderate
Isolation	Rubber dam is the standard of care; trauma makes isolation difficult, requiring adaptations (e.g., pre-endodontic build-ups).	Whether the choice of isolation method influences the clinical outcome of the barrier.	Expert opinion and case reports	Low
Irrigation	NaOCl is the gold standard; EDTA aids smear-layer removal and bioactive signaling.	Optimal concentration, volume, activation method, and definitive disinfection endpoint for wide, thin-walled canals.	Mainly laboratory (in vitro) data	Moderate
Intracanal Dressing	Calcium hydroxide is commonly used for disinfection; Triple antibiotic paste is an alternative.	Whether a dressing is always necessary; optimal material, duration, and necessity before apical plug placement.	Clinical studies with inconsistent findings	Low
Barrier Material	MTA, Biodentine, and bioceramic putties all show high clinical success (>85%).	Long-term superiority of newer pre-mixed putties over MTA/Biodentine; operator vs. material effect.	Short-term and retrospective clinical studies	Moderate
Barrier Thickness	A 4–5 mm apical plug is a clinically defensible, pragmatic target.	Optimal thickness depending on Cvek stage, restorative plan, and long-term fracture resistance.	Mainly laboratory data with limited clinical support	Low
Restoration	Coronal seal is as crucial as the apical seal; internal reinforcement (e.g., fibre posts) may prevent cervical fracture.	Best reinforcement method (e.g., custom posts, 3D-printed substructures); optimal final rinse after barrier set.	Mainly laboratory data	Low
Operator Experience	Likely an important factor influencing technical quality and treatment outcomes.	Causal confirmation; requires adjustment for case complexity in prospective studies.	Retrospective clinical data	Moderate

* Clinical Certainty: High = consistent clinical studies/guidelines; Moderate = clinical evidence with limitations; and Low = primarily laboratory, extrapolated, or expert opinion.

**Table 3:** Decision Boundary: Apical Barrier versus Regenerative Endodontic Procedures.

Factor	Regenerative Endodontics May Be Favoured	Apical Barrier May Be Favoured
Root Development	Very immature root, wide apex (Cvek stages II–III), with high potential for continued maturation and wall thickening.	More advanced immature root (Cvek stage IV), where predictable apical seal is prioritized over continued lengthening.
Infection Control	Controlled infection where bleeding can be induced to create a scaffold; absence of persistent purulent exudate.	Persistent infection, active exudate, or an uncertain regenerative environment where immediate sealing is required.
Restorability	Coronal structure allows for a staged, multi-visit regenerative protocol without compromising interim seal.	Extensive coronal breakdown requiring immediate obturation and/or intra-radicular reinforcement (e.g., fibre post).
Patient Compliance	Patient (and parent) can reliably attend multiple follow-up visits for monitoring and staged treatment.	Behavioural/time constraints or geographic inaccessibility favour a shorter, definitive one- to two-visit pathway.
Aesthetics	Use of non-staining materials/medicaments can be planned to avoid crown discolouration.	Material (e.g., MTA) selected carefully; coronal discolouration risk is managed, but aesthetics are secondary to preservation.
System/Context	Specialist endodontic access and reliable recall system are available for long-term monitoring.	Treatment delay is risky, limited specialist access, or recall is unreliable; requires a stable preservation endpoint.

**Table 4:** Consolidated Evidence Gaps and Future Research Priorities.

Priority	Evidence Gap/Unresolved Question	Current State of Evidence	Recommended Study Design to Address Gap
1. Standardised Outcome Reporting	Heterogeneity in case definitions, Cvek staging, follow-up duration, and conflation of success/healing/survival.	High heterogeneity; meta-analyses limited by non-comparable endpoints.	Consensus framework (e.g., Delphi process) for apexification outcomes; adequately powered RCTs with >5-year follow-up.
2. Disinfection & Endpoint	Optimal irrigant concentration, volume, and activation method; necessity/duration of intracanal dressings; microbiological verification of canal cleanliness.	Predominantly laboratory data; no clinical study has verified disinfection via bacterial sampling before plug placement.	Clinical trials with microbiological sampling (pre- and post-disinfection); comparison of single-visit vs. multi-visit dressings.
3. Barrier Thickness & Material Effect	Optimal thickness by Cvek stage/restorative plan; long-term superiority of newer putties over MTA/Biodentine; true operator vs. material effect.	Mainly laboratory data (thickness); short-term or retrospective clinical data (materials); operator effect confounded by case complexity.	Prospective RCTs stratified by Cvek stage and operator experience to isolate material and technique effects.
4. Definitive Restoration & Reinforcement	Best method for internal reinforcement (custom posts, 3D-printed substructures, polyethylene fibres); optimal final rinse after barrier set.	Largely confined to in vitro testing of standard fibre posts; no clinical studies linking reinforcement to long-term survival.	In vitro standardization followed by prospective clinical cohorts comparing reinforcement strategies in structurally compromised teeth.
5. Diagnostics & CBCT Protocol	Absence of a validated reference standard for pulp necrosis in immature teeth; selective indications for CBCT remain extrapolated from the trauma literature.	Extrapolated guidelines; no studies directly comparing CBCT vs. periapicals for altering apexification management.	Diagnostic accuracy studies comparing CBCT, periapicals, and new vitality tests (e.g., pulse oximetry) with histological or long-term clinical reference standards.

## Data Availability

No new data were created or analyzed in this study. Data sharing is not applicable to this article.
